# Nutritional composition and phytochemical screening in different parts of *Hibiscus syriacus* L.

**DOI:** 10.1002/fsn3.2899

**Published:** 2022-06-07

**Authors:** Yunmi Park, Soon‐ho Kwon, You Lim Jang, Doo‐Hee Lee, Seung‐Ok Yang, Hyun Ji Eo, Gwang Hun Park, Hae‐Yun Kwon

**Affiliations:** ^1^ Department of Forest Bio‐Resources National Institute of Forest Science Suwon South Korea; ^2^ 26725 National Instrumentation Center for Environmental Management Seoul National University Seoul South Korea; ^3^ Forest Medicinal Resources Research Center National Institute of Forest Science Yeongju South Korea

**Keywords:** antioxidant activity, functional food, *Hibiscus syriacus*, nutrition, phenolic compounds

## Abstract

As the national flower of Korea, the *Hibiscus syriacus* L. (Rose of Sharon) is symbolic in its abundance and is a prominent feature of Korean culture. *H. syriacus* has played an important role in Korea, not only as an ornamental plant but also as an essential ingredient in folk remedies through its various parts. This study aimed to characterize the nutritional and biochemical composition of each plant unit of *H. syriacus* “Wonhwa.” The units are namely: the petals, leaves, roots, and sprouts from its seeds. According to the results each unit produced, the sprouts had the highest content of amino acids and fatty acids which adhere to the requirements of nutritionally excellent food ingredients. The petals produced high quantities of glucose, sucrose, and fumaric acid, with the highest antioxidant activity among the four units. The main bioactive compounds detected in *H. syriacus* extracts in the four units were o‐coumaric acid, p‐coumaric acid, schaftoside, isoschaftoside, apigenin‐6‐C‐glucoside‐7‐o‐glucoside, and kaempferol‐3‐O‐galactoside‐7‐O‐rhamnoside. Overall, the highest number of bioactive compounds, 2 phenolic acids and 22 flavonoids, were identified in the petals. These results suggest the possibility of excellent pharmacological activity in the petals.

## Graphical abstract

This study was carried out with the intention of identifying benefits for human consumption of *H. syriacus* in terms of their individual units, as well as to detect other functional attributes of the components. To conclude, the results generated in this study strongly suggest the possibility of excellent pharmacological activity and nutritional advantages of not only the petals but across each division of the *H. syriacus* plant.

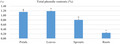

## 1 INTRODUCTION

*Hibiscus syriacus* L. (Rose of Sharon) is in abundance throughout Korea. It plays a significant role as the national flower in Korean culture. In addition to the ornamental and symbolic role that the flower fulfills, *H. syriacus* has been well known for its medicinal properties in traditional remedies. The biological make‐up of the tree classifies it as a part of the Malvaceae plant family and is widely grown in more than 50 countries around the world, including Europe and Asia (Kwon et al., 2017). Since *H. syriacus* can be grown in most regions, except for areas that experience extreme weather conditions, many *H. syriacus were* grown throughout Korea. More than 350 cultivars of *H. syriacus* were listed, and about 170 of these cultivars of *H. syriacus* were developed in Korea. The petals of these cultivars vary in color from white, pink, red, and purple, and the shape of the flower identifies as a single or double flower.

Since *H. syriacus* has beautiful flowers and a long flowering stage, it has often been grown in the garden or as a fence. And due to its availability and convenience, it is widely used as a food source at times of food scarcity in Korea, for example, steamed leaves as food and the addition of petals to rice cakes and tea (Bae, 1999). Furthermore, it is in demand due to its medicinal qualities; for example, its dried root and stem bark as antidotes, spring tonic, and fever reducers in traditional Korean remedies (Karunarathne et al., 2019).

Previous phytochemical investigations on this plant have resulted in the isolation of several naphthalenes, lignans, and flavonoids from root barks (Lee et al., 1999; Ryoo et al., 2010; Yoo et al., 1998). Recently, the antiproliferative activity of the root bark of this plant against cancer cells has been reported, and several triterpenoids were isolated as active constituents (Yeon et al., 2019). At present, there are many published studies on the pharmacological activity of the root and bark. Yet, there is less on the compounds and pharmacological effects of the other parts of the plant. Recently, two studies reported that the petal extracts of *H. syriacus* inhibit melanin, which is the cause of spots and freckles in the skin (Karunarathne et al., 2019). The young leaves of *H. syriacus* have also shown effects of in vitro anti‐inflammatory properties (Eo et al., 2020).

Although studies thus far have proven the pharmacological effects of *H. syriacus*, there has been no data comparing the nutritional value and phytochemical profiles in the different units for developing functional foods. The present research aimed to characterize the nutritional and biochemical composition of the sprouts, petals, roots, and leaves of *H. syriacus*, including their total polyphenol, antioxidative activity, and metabolic profile by LC‐MS. This study was carried out to identify benefits for human consumption of *H. syriacus* in terms of their individual units as well as to detect other functional attributes of the components.

## 2 MATERIALS AND METHODS

### 2.1 Plant material

This study was based on the acquired material of *H. syriacus* “Wonhwa” developed in 1990 by the National Institute of Forest Science in Korea. *H. syriacus* “Wonhwa” is a widely planted cultivar recommended by the Korea Forest Service in Korea, with large white petals and an excellent growth rate. The petals, roots, and leaves samples were collected from an 8‐year‐old tree of *H. syriacus* “Wonhwa” in an experimental site (37°15′04″N, 126°57′17″E) of the National Institute of Forest Science in mid‐August 2020. In the case of sprouts, naturally fruited seeds from another individual of the same cultivar in the same site were sown in mid‐April 2021 and cultivated for 15 days. All the samples collected were pulverized into powder form through a freeze‐drying process at −70°C immediately after harvesting.

### 2.2 Amino acid determination

One gram of freeze‐dried sample was weighed, and 30 ml of 80% methanol aqueous solution was used to extract these samples with sonication for 1 h. The extract was then filtered with a 0.2 μm membrane filter. The chromatic method were by the Agilent method (John et al., 2000). For derivatization of amino acids, the following reagents were used: 0.1 M borate buffer in water with 3‐mercaptopropionic acid (3‐MPA)/o‐phthalaldehyde (OPA) reagent and 9‐fluorenylmethyl chloroformate (FMOC‐Cl) reagent. Derivatization was performed using an automatic injector with a successive sampling of 5.0 μl of borate buffer and 1.0 μl of sample and then mixed. Subsequently, 1.0 μl of 3‐MPA/OPA reagent was added. After mixing, 1.0 μl of FMOC‐Cl was added and mixed, followed by 32 μl of water. Separations and analysis were performed with Dionex Ultimate 3000 (Thermo Dionex) equipped with an Agilent 1260 Infinity Fluorescence (FL) detector (Agilent). The chromatographic column was an Inno C18 column (4.6 mm × 150 mm, 5 µm, Youngjin Biochrom), at 40°C, with detection at λ = 340 nm.

The amino acid determination was performed with Mobile Phase A, consisting of 40 mM NaH2PO4, adjusted to pH 7.0, and Mobile phase, which was acetonitrile/methanol/water (45/45/10 v/v/v %), and a flow rate of 1.5 ml/min. The initial condition was 95% A of A for 3.0 min. 5% of B, followed by a linear gradient to 55%, 90% for 1 min, and 90% of B isocratically for 6 min. And then, washing and equilibration at 5% of B were performed.

To calculate the calibration curves, four different points were obtained using the amino acid standard mixture. The results are expressed in mg/100 g of dry weight.

### 2.3 Fatty acid determination

Samples of 100 mg with pentadecanoic acid (15:0) as the internal standard were placed in 4ml glass vials with Teflon caps. Derivatization was performed with 2ml of mixture solvent [methanol: benzene: 2.2‐dimethoxy‐propane (DMP): H2SO4 (39:20:5:2, by vol)] and heptane 1 ml at 80°C for 2 h incubation in a heating block. After heating, the tube was cooled to room temperature. Then, two phases formed. The upper phase containing FAMES was collected by GC (Agilent 7890A, Agilent). An Agilent DB‐23 (120 m × 0.25 mm × 0.25 µm) column was used with an injector temperature and FID detector temperature of 250 and 280°C, respectively, and a carrier gas of He. The split ratio was set to 10:1. The temperature program was as follows: 50°C for 1.0 min, increase at 25°C/min to 130°C, at 8°C/min to 170°C, at 1.5°C/min to 215°C, and at 5°C/min to 250°C for 5 min.

### 2.4 Free sugar determination

One gram of freeze‐dried sample and 30ml of 80% methanol aqueous solution were used to extract these samples with sonication for 1 h. The extract was filtered in a 0.2 μm membrane filter. The separations and analyses were performed with an HPLC system (Dionex ultimate 3000, Thermo Dionex), consisting of a Shodex Refractive Index (RI)‐101 detector (Shodex), a column heater set at 70°C, and a sugar‐pak column (300 × 6.5mm, Waters); the isocratic mobile phase was deionized‐distilled H2O delivered at 0.5 ml/min. The calibration curve with four different points was used to obtain the free sugar standard. The results are in mg/100 g of dry weight.

### 2.5 Organic acid determination

One gram of freeze‐dried sample was weighed. And a 30 ml of 80% methanol aqueous solution was used to extract these samples with sonication for 1 h. The extract was filtered with a 0.2 μm membrane filter. The HPLC system (Ultimate 3000, Thermo Dionex) with a pump system and an RI detector (ERC, RefractoMAX520) monitoring at 210 nm were used for organic acids analysis. Organic acids were simultaneously analyzed on an Aminex 87H column (300 × 10 mm, Bio‐Rad) at 40°C with a flow rate of 0.5ml/min and eluent of 0.01N H2SO4. Four different points were obtained using organic acid standards to calculate the calibration curves. The results are in mg/100 g of dry weight.

### 2.6 Total polyphenol content (TPC)

The extract was filtered with a 0.2 μm membrane filter. And 0.5 ml of distilled water was added to 100 μl of the extracted sample solution. Then, 100 μl of Folin & Ciocalteu phenol reagent was added and mixed. Subsequently, 1.0 ml of 7% Na2CO3 solution was added to the reaction solution. This mixed solution was allowed to stand at room temperature for 30 min. The absorbance was measured at 760 nm using a UV‐VIS spectrophotometer (Biomate5 spectrophotometer, Thermo Scientific). The total polyphenol content of the extracts was expressed as mg gallic acid equivalents (GAE) per gram of sample in dry weight.

### 2.7 ABTS radical cation scavenging activity

Ten grams of freeze‐dried sample was weighed. And 200 ml of 70% ethanol aqueous solution was used to extract these samples with sonication for 48 h. The ABTS assay was performed according to the method described by Li et al. (2009) with slight modification. The 2,2–azo–bis (3‐ethylbenzthiazoline‐6‐sulfonic acid) (ABTS, Sigma‐Aldrich) radical cation (ABTS+) was produced by mixing 7 mM ABTS solution with 2.45 mM potassium persulfate aqueous solution in the dark at room temperature for 16 to 24 h. The ABTS+ solution was diluted with phosphate buffer to the absorbance of 0.70 ± 0.02 at 732 nm (Perkin Elmer). Briefly, 90 μl ABTS solution and 10 μl of the sample were mixed and incubated at room temperature for 10 min. After 10 min, the absorbance was measured at 732 nm. For the blank, 10 μl DMSO was used instead of the sample. All tests were carried out in triplicate. The ABTS radical cation scavenging activity was calculated as follows:

ABTS radical cation scavenging activity (%) = [1 − (*Ai* − *Aj*) / A0] × 100, where A0 is the absorbance of the blank sample, *Ai* is the absorbance in the presence of the sample at different concentrations, and *Aj* is the absorbance of the blank reagent.

### 2.8 UHPLC‐TOF/HRMS analysis of phenolic compounds

0.1 g freeze‐dried sample was weighed. And 5 ml of 80% methanol aqueous solution was used to extract these samples with sonication for 1 h and filtered with a 0.2 μm membrane filter. An Ultimate 3000 RSLC UHPLC system (Thermo Fisher Scientific Inc.) was exposed to the phenolic compounds profiling of *H. syriacus* for the study. The system consisted of an autosampler, a column oven, an ultra‐high pressure solvent delivery pump, and an automatic degasser. Chromatographic separations of samples were performed using a Cortecs C18 column (150 × 2.1 mm, 1.6 μm, Waters Co.). The column temperature was set to 45°C. The injection volume was 5 μl with a flow rate of 0.3 ml/min. The mobile phases (0.1% formic acid in HPLC grade water, Solvent A; 0.1% formic acid in acetonitrile, Solvent B) was eluted with the linear gradient programmed as follows: (i) 0–1 min, 5% B; (ii) 1–10 min, from 5% to 30% B; (iii) 10–20 min, from 30% to 100% B; (iv) 20–24 min, 100% B; (v) 24–25 min 5% B. MS and MS/MS detection were conducted on a TripleTOF 5600+ (AB SCIEX, Concord) operating in a positive and negative electrospray ionization mode. The mass scan was used in full scan and information‐dependent acquisition (IDA) mode with the mass range set at 100–2000 m/z. The mass spectrometry conditions were set as follows: ion spray voltage 5.5 kV in positive mode and 4.5 kV in negative mode, ion source gas‐1 and gas‐2 50 psi, curtain gas pressure 35 psi, and the collision gas pressure 20 psi, source temperature 500°C, collision energy 35 ± 15eV in positive mode, and −35 ± 15 eV negative mode. The profiling of phenolic compounds was identified based on their accurate mass (m/z) and molecular (m/z) ion fragmentation pattern using Scafford 2,2,1, and several mass spectral libraries such as NIST Library 2017 and MassBank of North America (MoNA) library database.

### 2.9 Statistical analysis

Triplicate analyses for each plant unit (leaves, petals, roots, and sprouts) were carried out. The obtained results were statistically processed using the program package SPSS 20. Statistical significance was considered at probability *p* < .01 based on Duncan's new multiple range test calculators. The mean values and standard deviations (SD) were calculated for all analyzed compounds. The data were as mean ± SE.

## 3 RESULTS AND DISCUSSION

### 3.1 Amino acid and fatty acid composition

Total amino acid contents of *H. syriacus* extracts from its different units (leaves, petals, sprouts, and roots) results from the test are presented in Table 1. Of a total of 20 amino acids, all but cysteine, 19 amino acids were isolated, including the ten essential amino acids. The sum of amino acid content within each unit was highest in the sprouts (1274.6 mg/100 g), followed by the petals (737.2 mg/100 g), roots (581.5 mg/100 g), and leaves (277.1 mg/100 g). The total amino acid content of *H. syriacus* sprouts grown for 10 days was 1274.6 mg/100 g, lower than that of radish sprouts (*Raphanus sativus* L., 3020 mg/100 g) grown for 12 days, but produced a similar value to the 1313.1 mg/100 g from barley sprouts (*Hordeum vulgare* L.), a popular source for health food in Korea (Han et al., 2003; Son et al., 2016), grown for 10 days. To be used as food in the form of sprouts of *Hibiscus syriacus*, it should be superior in other excellent ingredients and amino acid content to the existing sprouts vegetables (radish, barley sprouts, and so on).

**TABLE 1:** Amino acid composition of each unit off *H. syriacus* (Unit: mg/100 g)

	Leaves	Petals	Roots	Sprouts
Aspartic acid	37.0 ± 1.2^c^	33.1 ± 0.5^bc^	26.7 ± 1.8^a^	29.3 ± 0.8^ab^
Glutamic acid	70.7 ± 1.6^c^	31.0 ± 0.5^b^	19.5 ± 1.1^a^	**87.5 ± 0.6** ^d1^
Asparagine	8.7 ± 0.4^a^	160.0 ± 2.2^b^	157.4 ± 7.2^b^	**268.1 ± 8.3^c^**
Serine	21.6 ± 0.5^b^	63.4 ± 0.8^c^	9.9 ± 0.5^a^	**147.7 ± 1.4^d^**
Glutamine	21.6 ± 0.4^a^	**101.2 ± 0.7^d^**	24.7 ± 0.8^b^	47.1 ± 0.5^c^
**Histidine**	1.3 ± 0.4^a^	8.0 ± 0.5^b^	**18.9 ± 1.5^d^**	13.5 ± 1.0^c^
Glycine	7.9 ± 0.3^b^	39.8 ± 0.3^c^	2.2 ± 0.1^a^	**295.3 ± 2.5^d^**
**Threonine**	12.4 ± 0.3^b^	25.3 ± 0.1^c^	7.3 ± 0.3^a^	**46 ± 0.8^d^**
**Arginine**	7.3 ± 0.4^a^	1.7 ± 0.1^a^	**274.4 ± 9.6^c^**	134.6 ± 1.6^b^
Alanine	44.7 ± 0.7^b^	**171.6 ± 1.4^d^**	4.1 ± 0.3^a^	136.9 ± 2.4^c^
Tyrosine	2.7 ± 0.1^b^	3.2 ± 0.1^b^	**7.2 ± 0.2^c^**	1.6 ± 0.2^a^
**Valine**	7.8 ± 0.2^b^	**22.6 ± 0.1^d^**	2.4 ± 0.1^a^	12.9 ± 0.2^c^
**Methionine**	0.5 ± 0.1^b^	**1.6 ± 0.1^c^**	0.1 ± 0.0^a^	0.4 ± 0.1^ab^
**Tryptophan**	9.1 ± 0.4^ab^	9.3 ± 0.1^b^	8.3 ± 0.3^a^	**12.1 ± 0.1^c^**
**Phenylalanine**	3.8 ± 0.2^b^	**14.5 ± 0.2^d^**	1.5 ± 0.1^a^	6.5 ± 0.5^c^
**Isoleucine**	5.2 ± 0.3^b^	**10.5 ± 0.1^d^**	1.3 ± 0.0^a^	8.5 ± 0.3^c^
**Leucine**	5.9 ± 0.2^b^	5.9 ± 0.0^b^	1.2 ± 0.0^a^	**6.9 ± 0.1^c^**
**Lysine**	5.4 ± 0.2^c^	5.0 ± 0.6^bc^	2.2 ± 0.2^a^	4.2 ± 0.1^b^
Proline	3.6 ± 0.8^a^	**29.3 ± 1.4^c^**	12.3 ± 0.9^b^	15.7 ± 1.1^b^
Total EAA^2^	58.7 ± 3.6	104.5 ± 8.1	317.6 ± 85.5	245.4 ± 40.6
Total AA^3^	277.1 ± 18.1	737.2 ± 51.1	581.5 ± 68.6	1274.6 ± 90.1
EAA/ TAA^4^	21.2%	14.2%	54.6%	19.2%

Note

(1) The data are presented as mean values ±standard error (*n* = 3); superscript lowercases (a–d) indicate homogenous subclasses as resulted from ANOVA (*p* < .05, Duncan's new multiple range test) among units.

(2) Total EAA: Total essential amino acid.

(3) Total AA: Total amino acid.

(4) EAA/TAA: Essential amino acid/total amino acid.

Bold letters mean the essential amino acid.

Among the 19 amino acids, seven amino acids, including alanine (171.6 ± 1.4 mg/100 g) and glutamine (101.2 ± 0.7 mg/100 g), were the highest content in the petals; these, also including glycine (295.3 ± 2.5 mg/100 g) and asparagine (268.1 ± 8.3 mg/100 g), yielded the highest in the sprouts (*p* < .01). Among the remaining amino acids, histidine, arginine, and tyrosine were the most abundant in the roots compared to the other units (*p* < .01). The essential amino acid content was high in the roots, at 317.6 mg/100 g, and sprouts, at 245.4 mg/100 g, with a significantly higher production of amino acids; arginine, at 274.4 mg/100 g, 134.6 mg/100 g, respectively. These results are significantly higher than radish sprouts (*Raphanus sativus* L, 120.7 mg/100 g) used as an ingredient in bibimbap, a traditional Korean food (Han et al., 2003). Arginine, which is converted into nitric oxide and citrulline in the body, improves circulation in the bloodstream. It acts as a vasodilator (Dong et al., 2011). Currently, many healthy functional foods containing arginine have been developed. It is worth examining whether the root, with the highest arginine content among the four parts, can be used for healthy food.

Table 2 shows the total contents of the four fatty acids distributed by each unit as follows: sprouts (1070.0 mg/100 g), leaves (821.6 mg/100 g), roots (407.0 mg/100 g), petals (369.1 mg/100 g). The sum of fatty acid content was as follows: linoleic acid (1311.2 mg/100 g), α‐linolenic acid (663.1 mg/100 g), palmitic acid (592.6 mg/100 g), oleic acid (100.8 mg/100 g). Also, the ratio of unsaturated fatty acids (linoleic acid, α‐linolenic acid, oleic acid) to saturated fatty acids (palmitic acid) was 77.8%–22.2% in total. Among the four types of fatty acids, except for α‐linolenic acid, the sprouts had the highest content compared to the other units (*p* < .01). In *Actinidia argute* leaves, used as a spring vegetable in Korea, α‐linolenic acid accounted for 66.8% of the total fatty acid content, linoleic acid was 13.8% as an unsaturated fatty acid, and palmitic acid accounted for 12.1% (Jin et al., 2015). In the flowers of *Robinia pseudoacacia* L., popular as an edible flower in Korea, palmitic acid represented 44.4% of the saturated fatty acids, followed by linoleic acid at 19.3% and α‐linolenic acid at 12.9% of the unsaturated fatty acids. On the other hand, in this study, palmitic acid represented 30.9%, then, followed by 69.1% of the unsaturated fatty acids, including linoleic acid, α‐linolenic acid, and oleic acid in petals of *H. syriacus*.

**TABLE 2:** Fatty acid composition of each unit off *H. syriacus* (Unit: mg/100 g)

	Leaves	Petals	Roots	Sprouts	Sum
Palmitic acid	166.2 ± 1.9^c^	114.2 ± 1.1^b^	89.5 ± 1.0^a^	**222.7 ± 1.7^d1^**	592.6 ± 2.8
Oleic acid	0 ± 0^a^	17.7 ± 0.2^b^	18.9 ± 0.3^c^	**64.1 ± 0.8^d^**	100.8 ± 0.7
Linoleic acid	339 ± 3.8^c^	181.3 ± 2^a^	264.4 ± 3.1^b^	**526.5** ± **4^d^**	1311.2 ± 7.8
α‐Linolenic acid	**316.5 ± 3.5^d^**	55.8 ± 0.4^b^	34.1 ± 0.6^a^	256.7 ± 1.5^c^	663.1 ± 1989.3
Saturated fatty acid (%)	20.2	30.9	22.0	20.8	22.2
Unsaturated fatty acid (%)	79.8	69.1	78.0	79.2	77.8
Sum	821.6 ± 8.8	369.1 ± 3.6	407.0 ± 5.0	1070.0 ± 7.8	2667.6 ± 14.6

Note

(1) The data are presented as mean values ±standard error (*n* = 3); superscript lowercases (a–d) indicate homogenous subclasses as resulted from ANOVA (*p* < .05, Duncan's new multiple range test) among units.

*H. syriacus* sprouts had the highest content of amino and fatty acids and can provide adequate data for future development as an ingredient in the food. Moreover, since *H. syriacus* blooms new flowers every day for about 100 days, the seed yield is higher than other flowering trees. It will be easy to obtain the sprouts by cultivating these seeds within a short period.

### 3.2 Free sugars and organic acids composition

The total free sugars contents of *H. syriacus* extracts from distinct parts of the plant (leaves, petals, sprouts, and roots) were tested with the data content presented in Table 3. The sum of the free sugar content within each unit was as follows: petals (9141.1 mg/100 g), leaves (2413.3 mg/100 g), sprouts (1429.3 mg/100 g), and roots (707.8 mg/100 g). The leaves contained five types of free sugars, while the petals had three, the sprouts two, and the roots one. The leaves contained glucose (847.9 mg/100 g) and raffinose (836.3 mg/100 g) presented similarly high values. Trisaccharide raffinose and tetrasaccharide stachyose were detected only in the leaves.

**TABLE 3:** Free sugar composition of each unit of *H. syriacus* (Unit: mg/100 g)

	Leaves	Petals	Roots	Sprouts	Sum
Fructose	56.6 ± 3.4	4085.0 ± 17.5	‐	‐	4141.7 ± 14.6
Glucose	847.9 ± 4.9	3873.8 ± 25.7	‐	949.5 ± 15.4	5671.3 ± 36.6
Raffinose	836.3 ± 2.4	‐	‐	‐	836.3 ± 2.4
Stachyose	135.1 ± 7.3	‐	‐	‐	135.1 ± 7.3
Sucrose	537.3 ± 4.8^b^	**1182.2 ± 3.7^d1^**	707.8 ± 8.8^c^	479.8 ± 9.7^a^	2907.2 ± 19.9
Sum	2413.3 ± 11.7	9141.1 ± 38.1	707.8 ± 8.8	1429.3 ± 22.4	‐

Note

(1) The data are presented as mean values ±standard error (*n* = 3); superscript lowercases (a–d) indicate homogenous subclasses as resulted from ANOVA (*p* < .05, Duncan's new multiple range test) among units.

The petals contained high levels of fructose (4085.0 mg/100 g), glucose (3873.8 mg/100 g), and sucrose (1182.3 mg/100 g). The highest content of glucose and sucrose found in the petals was at (*p* < .01). The roots contained only sucrose (707.8 mg/100 g), and the sprouts contained both glucose (949.5 mg/100 g) and sucrose (479.8 mg/100 g). Although the free sugar content in *H. syriacus* petals was lower than that of *Robinia pseudoacacia* L. flowers (fructose 7500.2 mg/100 g, glucose 300.7 mg/100 g, sucrose 6100.7 mg/100 g), it was higher than that of *Rhododendron mucronulatum* Turcz. These are considered edible flowers during the spring season in Korea (Kwon et al., 1995).

Table 4 shows the total organic acid content of each part of *H. syriacus*. The organic acid content of each part was as follows: leaves (524.9 mg/100 g), sprouts (508.0 mg/100 g), petals (460.9 mg/100 g), and roots (186.4 mg/100 g). The organic acids identified in *H. syriacus* were citric acid, fumaric acid, and malic acid. The leaves and sprouts contained all three organic acids, while the petals contained only fumaric acid, and the roots contained citric acid and fumaric acid. Citric acid, a well‐known natural ingredient extracted from sour fruits such as lemon and lime, is widely used as a preservative and is evenly contained in the leaves, roots, and sprouts of this plant, with the highest yield of 241.6 mg/100 g, found in the sprouts (Owen et al., 2002). The fumaric acid is found in each of the units, the leaves, petals, roots, and sprouts of *H. syriacus*, with the highest aggregate of 460.9 mg/100 g in petals. In comparison, the highest contents were those of fumaric acid (822.2 mg/100 g), citric acid (646.5 mg/100 g), and malic acid (211.5 mg/100 g). Fumaric acid, and its esters, possess the following: anti‐inflammatory, hepatoprotective, analgesic, antitumor, and anti‐intoxication activities, as well as strong antibacterial activity against *Staphylococcus aureus, Streptococcus*, *Escherichia coli*, and *Salmonella* (He et al., 2011; Shakya et al., 2014).

**TABLE 4:** Organic acid composition of each unit of *H. syriacus* (Unit: mg/100 g)

	Leaves	Flowers	Roots	Sprouts	Sum
Citric acid	229.7 ± 4.0^b^	‐	175.3 ± 1.8^a^	**241.6 ± 3.7^c1^**	646.5 ± 2.3
Fumaric acid	135.7 ± 1.2^b^	**460.9 ± 3.6^d^**	11.2 ± 0.3^a^	214.4 ± 6.1^c^	822.2 ± 9.3
Malic acid	159.5 ± 5.0^*^	‐	‐	52.0 ± 1.5	211.5 ± 5.0
Sum	524.9 ± 1.9	460.9 ± 3.6	186.4 ± 1.5	508.0 ± 4.5	

Note

(1) The data are presented as mean values ±standard error (*n* = 3); superscript lowercases (a–d) indicate homogenous subclasses as resulted from ANOVA (*p* < .05, Duncan's new multiple range test) among units.

*H. syriacus* blooms about 50–100 flowers per tree each day from early July to mid‐October, that is, for nearly 100 days, offering the advantage of a long duration of use compared to other climate‐sensitive and seasonal plants. In this study, the contents of fructose and sucrose, which give a sweet taste, and fumaric acid were highest in petals. Those results show an advantage when used as an edible flower and medicinal herb.

### 3.3 Total polyphenols content and antioxidant capacity

**FIGURE 1:**
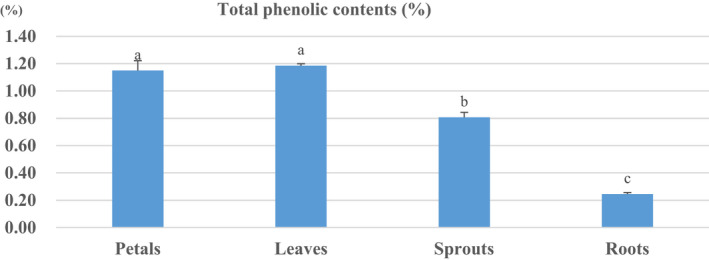
Total phenolic contents within the different units of *H. syriacus*. The data are presented as mean values ±standard error (*n* = 3); superscript lowercases (a–c) indicate homogenous subclasses as resulted from ANOVA (*p* < .05, Duncan's new multiple range test) among units

The result of the analysis of total polyphenol content of *H. syriacus* extracts within its different units (leaves, petals, roots, and sprouts) is in Figure 1. The phenolic compounds are secondary metabolites, widely distributed in plants systems, having various functions exhibiting a physiologically active functionality as an antioxidant. It is due to its strong binding properties to proteins and other macromolecules (AOAC, 1995). Among the total polyphenolic content of *H. syriacus*, leaves (1.18 ± 0.01%) and petals (1.15 ± 0.07%) showed the highest total value compared to the value of the sprouts (0.81 ± 0.04%) and roots (0.24 ± 0.01%).

**FIGURE 2:**
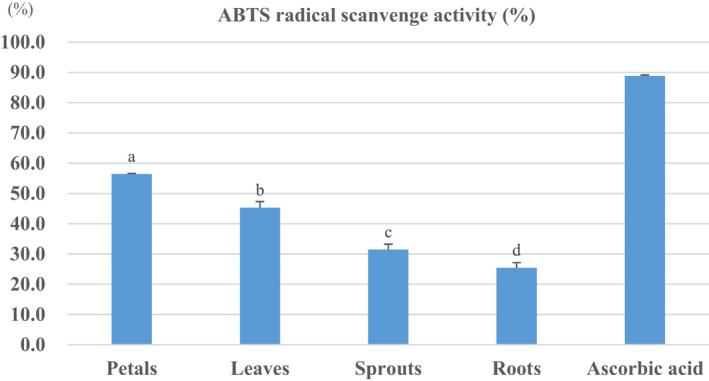
ABTS free radical scavenging assays within the different units of *H. syriacus*. The data are presented as mean values ±standard error (*n* = 3); superscript lowercases (a–d) indicate homogenous subclasses as resulted from ANOVA (*p* < .05, Duncan's new multiple range test) among units

The 70% EtOH extracts of each *H. syriacus* unit showed high antioxidant activity in ABTS free radical scavenging assays. At a low concentration of 25 μg/ml, the antioxidant activity within each unit was as follows: the highest value established at 56.4% for petals, 45.3% for leaves, 31.4% for sprouts, and 25.4% for roots, respectively (Figure 2). Following these results, it is understood that the antioxidant activity was high in the petals with the highest polyphenol content. The calyx fruits of *Hibiscus sabdariffa* L., which is a plant of considerable medicinal and economical value worldwide as a genus of *Hibiscus*, showed less than 40% free radical scavenging activity in hot water extract and 30%–95% in EtOH extract at 200μg/mL which is a higher concentration (Yang et al., 2012).

### 3.4 Identification of phenolic compounds by UHPLC‐TOF/HRMS

A targeted analysis, based on UHPLC‐TOF/HRMS (ultra‐high‐performance liquid chromatography–time‐of‐flight high‐resolution mass spectrometry), was conducted to identify the phenolic compounds present in methanolic extracts of the units of *H. syriacus* since the phenolic composition of *H. syriacus* is still not reported in the literature. Table 5 shows that the phenolic profile was diverse among the leaves, petals, roots, and sprouts of *H. syriacus*. The six compounds detected in extracts of each *unit* of *H. syriacus* were o‐coumaric acid, p‐coumaric acid, schaftoside, isoschaftoside, Apigenin‐6‐C‐glucoside‐7‐O‐glucoside (saponarin), and Kaempferol‐3‐O‐galactoside‐7‐O‐rhamnoside. O‐coumaric acid shows anticarcinogenic and anti‐obesity effects in in vitro cell culture system (natural products). P‐coumaric acid, identified in the leaf and flower extracts of *Hibiscus roseus*, possess the following: depigmentation, anti‐inflammatory, and tyrosinase inhibition activities with potential skin cosmetic applications (Boo, 2019; Sen et al., 2013; Song et al., 2011; Varela et al., 2020). Schaftoside and isoschaftoside, a pair of flavonoid di‐C‐glycosides, possess a variety of biological activities, including the following: anti‐respiratory syncytial virus, anti‐diabetic, antihypertensive, hepatoprotective, anti‐inflammatory, and antioxidant activities to mammals, indicating their potential applications as drugs or dietary supplements (Wang et al., 2020). Saponarin (Apigenin‐6‐C‐glucoside‐7‐O‐glucoside), a natural flavonoid, is known for its antioxidant and hepatoprotective properties found in a diverse number of plants, including *Tinospora cordifolia*, used as an anti‐diabetic drug, and barley sprouts (Sengupta et al., 2009; Son et al., 2016). Kaempferol 3‐O‐galactoside‐7‐O‐rhamnoside is a member of the class of compounds known as flavonoid‐7‐o‐glycosides, and numerous preclinical studies have shown that kaempferol and some glycosides of kaempferol have a wide range of pharmacological attributes including, but not limited to, antioxidant, anti‐inflammatory, antimicrobial, and anticancer subscriptions (Calderon‐Montano et al., 2011). Interestingly, phenolic acid and flavonoid compounds contained in the different units of *H. syriacus* were not previously reported. Their identification in the leaf and flower extracts of *Hibiscus roseus co*uld encourage further investigation of promising sources of natural, biological, and active molecules. With the six compounds identified within the units, it would be advisable to select the part of *H. syriacus* that provides the most nutritional content through quantitative analysis to use *H. syriacus* for developing functional food and natural drugs.

**TABLE 5:** Distribution of (tentatively) Identified Metabolites in Different Units of *H. syriacus*

	R.T (min)	Analyte name	Molecular formula	Molecular weight	Adduct	Mass error (ppm)			
						Leaves	Petals	Roots	Sprouts
1	5.36	Isoorientin 2''‐O‐glucoside	C27H30O16	610.1534	[M + H]^+^	‐	1.933	‐	‐
2	5.86	Isoorientin 2''‐O‐rhamnoside	C27H30O15	594.1585	[M + H]^+^	‐	1.831	‐	‐
3	5.93	Luteolin 8‐C‐glucoside	C21H20O11	448.1006	[M + H]^+^	‐	2.715	‐	‐
4	6.3	o‐Coumaric acid	C9H8O3	164.0473	[M + H−H2O]^+^	2.558	2.627	2.88	1.411
5	7.05	p‐Coumaric acid	C9H8O3	164.0473	[M + H − H2O]^+^	2.138	2.233	1.802	1.954
6	7.19	Catechin	C15H14O6	290.0790	[M − H]−	‐	2.357	‐	‐
7	7.65	Luteolin 4'‐glucoside	C21H20O11	448.1006	[M + H]^+^	‐	1.168	‐	‐
8	8.41	1‐O‐feruloyl‐β‐D‐glucose	C16H20O9	356.1107	[M − H]−	2.631	‐	‐	‐
9	8.56	Kaempferol 3,7‐diglucoside	C27H30O16	610.1534	[M − H]−	1.446	1.266	‐	‐
10	9.16	Quercetin‐3‐O‐b‐glucosyl‐7‐O‐a‐rhamnoside	C27H30O16	610.1534	[M − H]−	‐	1.83	‐	‐
11	9.33	Luteolin‐7,3'‐di‐O‐glucoside	C27H30O16	610.1534	[M − H]−	‐	1.507	‐	‐
12	9.42	Schaftoside	C26H28O14	564.1479	[M − H]−	2.425	2.06	2.056	2.683
13	9.49	Apigenin‐6‐C‐glucoside‐7‐O‐glucoside	C27H30O15	594.1585	[M + H]−	1.078	1.74	0.512	1.257
14	9.66	Myricetin‐3‐Galactoside	C21H20O13	480.0904	[M − H]−	‐	2.339	‐	‐
15	9.81	Isoschaftoside	C26H28O14	564.1479	[M − H]−	1.144	1.201	1.421	0.699
16	10.44	Kaempferol‐3‐O‐galactoside‐7‐O‐rhamnoside	C27H30O15	594.1585	[M − H]−	1.374	2.304	2.089	1.858
17	10.51	Quercetin‐3‐O‐robinobioside	C27H30O16	610.1534	[M − H]−	2.008	‐	‐	1.423
18	10.59	2‐O‐Rhamnosylvitexin	C27H30O14	578.1636	[M − H]−	1.477	1.437	‐	‐
19	11	Sorbifolin 6‐O‐β‐glucopyranoside	C22H22O10	446.1213	[M + H]^+^	0.532	1.35	‐	1.421
20	11.29	Quercetin‐3‐O‐glucose‐6''‐acetate	C23H22O13	506.1060	[M − H]−	2.644	2.011	‐	1.453
21	11.37	Astragalin	C21H20O11	448.1006	[M − H]−	‐	1.522	‐	‐
22	11.9	6‐C‐Glucosyl‐8‐C‐rhamnosylapigenin	C27H30O14	578.1636	[M + H]^+^	‐	1.732	‐	‐
23	11.91	Naringenin‐7‐glucoside	C21H22O10	434.1213	[M + H]^+^	‐	1.362	‐	‐
24	12.01	Naringenin	C15H12O5	272.0685	[M + H]^+^	‐	0.855	‐	‐
25	12.08	Apigetrin	C21H20O10	432.1056	[M + H]^+^	0.642	1.94	‐	‐
26	14.67	Kaempferol‐7‐O‐rhamnoside	C21H20O10	432.1056	[M + H]^+^	‐	1.679	‐	‐

Among the four units of *H. syriacus* observed during the research process, the most flavonoids were in the petals. The main compound classes only detected in white petals of *H. syriacus* extracts were: Isoorientin derivatives, Luteolin derivatives, Catechin, Quercetin‐3‐O‐b‐glucosyl‐7‐O‐a‐rhamnoside, 6‐C‐glucosyl‐8‐C‐rhamnosylapigenin, Myricetin‐3‐galactoside, Astragalin, Naringenin, Naringenin‐7‐glucoside, and Kaempferol‐7‐O‐rhamnoside. Various pharmacological effects of these flavonoids have been revealed. It is then necessary to recognize the exact content in flowers through quantitative analysis. A recently published paper on the pharmacological effects of white petals of *H. syriacus* is the inhibition of melanin production and osteoclast inhibition differentiation that causes osteoporosis (Karunarathne et al., 2019; Lee et al., 2015).

One compound only detected in the leaves of *H. syriacus* extracts was 1‐O‐feruloyl‐β‐D‐glucose, a phenolic compound isolated from the corks of *Euonymus alatus* (Thunb. Sieb) and showed an anti‐adipogenic effect on 3T3‐L1 preadipocytes in previous studies (Kwak & Kim, 2018). The compound detected solely in *H. syriacus* extracts of leaves and sprouts was quercetin‐3‐O‐robinobioside. The main classes of compounds detected in *H. syriacus* extracts of leaves and petals were sorbifolin 6‐O‐β‐glucopyranoside and quercetin‐3‐O‐glucose‐6''‐acetate, excluding the roots and stem. The main classes of compounds distinguished within the *H. syriacus* extracts of leaves and petals were Kaempferol 3,7‐diglucoside, 2‐O‐Rhamnosylvitexin, and Apigetrin. Recent reports showed kaempferol 3,7‐diglucoside in the leaf extracts of *Evolvulus alsinoides* (Linn.) with inhibition potency against α‐amylase, α‐glucosidase, acetylcholinesterase, and amyloid aggregation (Sundaramoorthy & Packiam, 2021). Apigetrin, a flavonoid found in many plant leaves and seeds, has been known to possess antimutagenic, anticancer, antioxidant, and anti‐inflammatory properties (Patel & Patel, 2021). Overall, 2 phenolic acids and 22 flavonoids were identified in the petals, which yielded the highest number compared to other units.

## 4 CONCLUSION

This study highlights the abundance in the nutritional composition of each of the four units of *H. syriacus* commonly cultivated in Korea. *H. syriacus* sprouts contain amino and fatty acids, which are of the highest aggregate in identifying their value as a functional food, and, since seeds are the means of propagation, sprouts are produced in abundance. *H. syriacus* petals contain a large number of free sugars, including fructose and sucrose, along with fumaric acid, which is why they are the most valuable as edible flowers or healthy functional foods having the highest number compared to the other parts. Subsequently, 2 phenolic acids and 22 flavonoids, were also identified in the petals.

To conclude, the results generated in this study strongly suggest the possibility of excellent pharmacological activity and nutritional advantages, not only in the petals but across each division of the *H. syriacus* plant.

## CONFLICT OF INTEREST

The author declares no conflict of interest.

## Data Availability

The data used to support the findings of this study are included within the article.
